# Δ^9^-Tetrahydrocannabinol (THC) and Cannabidiol (CBD) Diminish CD16^+^ Monocyte-Induced Astrocyte Inflammation, while THC Uniquely Inhibits Monocyte Chemotaxis Independent of HIV Status

**DOI:** 10.1007/s11481-026-10300-2

**Published:** 2026-07-04

**Authors:** Sera Sermet, Robert B. Crawford, Peter Gulick, Norbert E. Kaminski

**Affiliations:** 1 Department of Pharmacology and Toxicology, Michigan State University, 1129 Farm Lane, Rm. 165G, Food Safety Toxicology Building, East Lansing, MI 48824 USA; 2 Institute for Integrative Toxicology, Michigan State University, East Lansing, MI 48824 USA; 3 Department of Osteopathic Medicine, Michigan State University, East Lansing, MI 48824 USA; 4 Center for Research on Ingredient Safety, Michigan State University, East Lansing, MI 48824 USA

**Keywords:** Δ^9^-tetrahydrocannabinol (THC), Cannabidiol (CBD), Neuroinflammation, Chemotaxis, Monocyte, Astrocyte, HIV

## Abstract

CD16^+^ monocytes are a minor subset of the total monocyte population that play a disproportionate role in contributing to neuroinflammation in human immunodeficiency virus (HIV)-associated neurocognitive disorders (HAND). This has been evidenced by the enhanced transmigration of CD16^+^ monocytes into the brain compared to their CD16^−^ counterpart. CD16^+^ monocytes can be activated by HIV ssRNAs through toll-like receptors (TLR) 7 and TLR8, and subsequently interact with brain-resident cells, including astrocytes. Previous studies from our laboratory identified monocyte-derived IL-1ß as an inducing cytokine for astrocyte-derived neuroinflammatory factors. Despite cannabis use among the HIV community, the mechanisms by which immune-modulating cannabinoids, Δ^9^-tetrahydrocannabinol (THC) and cannabidiol (CBD), alter human immune responses in the context of HAND-associated neuroinflammation remain elusive. We hypothesized that THC and CBD suppress CD16^+^ monocyte-induced astrocyte secretion of inflammatory mediators and monocyte recruitment via chemotaxis in the context of HIV. Results from this study show that THC and CBD impair CD16^+^ monocyte IL-1ß-mediated astrocyte production of IL-6, IL-8, and MCP-1 when these two cell types are cocultured in the presence of TLR7 or TLR8 stimulation. Additionally, monocytes from HIV+ subjects exhibited enhanced migration compared to monocytes from HIV- subjects, which was suppressed by THC treatment but not by CBD. The effects on migration were associated with reduced cellular expression of polymerized actin and high-affinity conformation integrin receptors. Collectively, these findings suggest that THC, and to a lesser extent CBD, may have therapeutic potential for mitigating CD16^+^ monocyte-mediated neuroinflammation associated with HAND.

**Graphical Abstract:**

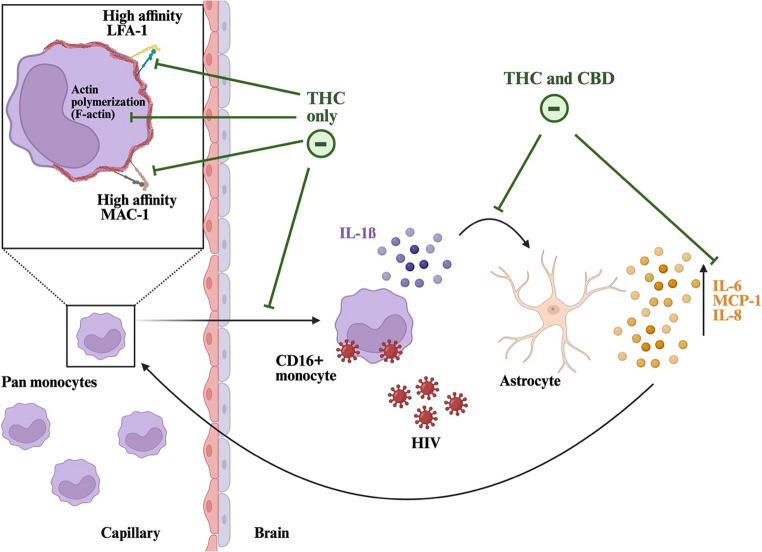

## Introduction

Neuroinflammation is a hallmark of central nervous system (CNS) diseases and can be triggered by dysfunction of the blood-brain barrier (BBB), which enables peripheral immune cell infiltration (Hinz and Geschwind 2017; Shi and Yong 2025). Furthermore, the brain possesses resident immune cells, such as astrocytes, which are the most abundant glial cell type in the CNS. Astrocytes serve as immune regulators of the brain with the primary function of protecting neural tissue, maintaining neural homeostasis, and preserving neurological function following CNS injuries (Colombo and Farina 2016; Fisher and Liddelow 2024; Priego and Valiente 2019). While the CNS is known to have active immune surveillance and neuroimmune signaling, which serve essential functions for maintaining homeostasis and protection against brain injury, dysregulation of this tightly controlled microenvironment can result in pathological consequences, such as neuroinflammation (Spath et al. 2017). Peripheral immune cell infiltration into the CNS and the resulting neuroinflammation can damage brain-resident cells, ultimately contributing to neurodegeneration (Netzahualcoyotzi et al. 2024; Spath et al. 2017). Furthermore, monocyte invasion into the CNS has been implicated in several neurodegenerative conditions, such as Human Immunodeficiency Virus (HIV)-Associated Neurocognitive Disorder (HAND), multiple sclerosis, and Alzheimer’s disease (Clay et al. 2007; Fischer-Smith et al. 2001; Muñoz-Castro et al. 2023; Tian et al. 2025).

Monocytes are a heterogeneous population of innate immune cells that can be defined by their surface expression of markers CD14 and CD16. Furthermore, monocytes are subcategorized into three distinct populations: classical monocytes (CD14^+^CD16^−^), intermediate monocytes (CD14^+^CD16^+^), and non-classical monocytes (CD14^dim^CD16^++^) (Bernward Passlick 1989; Ziegler-Heitbrock et al. 2010). CD16^+^ monocytes are a minor subset of the total monocyte population, comprised of both intermediate and non-classical monocytes. They account for approximately 10% of the total monocyte pool (Bernward Passlick 1989; Burbano et al. 2014). CD16^+^ monocytes are characterized by their release of inflammatory mediators, such as interleukin-1ß (IL-1ß), and tissue extravasation through chemotaxis, which allows for directional movement of a cell toward a site of infection or inflammation (Cros et al. 2010; Luger et al. 1983; Serbina and Pamer 2006; Sermet et al. 2025; Wong et al. 2011). Chemotaxis relies on adhesion to halt cellular motion using surface integrin receptors such as lymphocyte function-associated antigen-1 receptor (LFA-1) and macrophage-1 antigen receptor (MAC-1) (Li et al. 2018; Min et al. 2005; Schenkel et al. 2004; Sumagin et al. 2010). Integrin receptors undergo dynamic conformational changes from a low-affinity closed state to a high-affinity open state to enable ligand binding, thus are not persistently ready to initiate effective adhesion (Li et al. 2017; Takagi et al. 2002). Additionally, directed cellular migration toward a chemotactic gradient requires tightly regulated cytoskeletal reorganization of actin polymerization, whereby globular actin (G-actin) assembles into filamentous actin (F-actin) (Hwang et al. 2024; Weiner et al. 1999). These rapid cytoskeletal dynamics are essential for establishing and sustaining directional monocyte chemotaxis.

Peripheral CD16^+^ monocytes have been specifically identified to traffic to the brain, where they can contribute to neuroinflammation associated with HAND (Clay et al. 2007; Fischer-Smith et al. 2001). Furthermore, the CNS is considered to be a viral reservoir for latent HIV despite antiretroviral therapy, particularly due to the limited BBB penetrance of antiretroviral therapeutics (Joseph et al. 2018; Osborne et al. 2020; Suzuki et al. 2022). Clinical studies report that in some individuals with successful peripheral viral suppression, viral RNA remains detectable in the cerebrospinal fluid despite antiretroviral intervention, indicating compartmentalized viral escape (Joseph et al. 2018; Peluso et al. 2012). Moreover, studies show that monocytes can recognize HIV ssRNAs via intracellular toll-like receptors (TLRs)7 and TLR8, leading to their activation and production of inflammatory cytokines, including IL-1ß, IL-6, and tumor-necrosis factor-α (TNF-α) (Guo et al. 2014; Heil et al. 2004). The production of these inflammatory factors by monocytes in the CNS can cause neuronal injury and astrocyte dysfunction, resulting in compromised BBB integrity (Argaw et al. 2006; Chen et al. 2022; Rossi et al. 2014). Consistent with this, previous work from our laboratory has demonstrated that in a human coculture system containing primary pan monocytes and primary fetal astrocytes, neutralization of monocyte-derived IL-1ß largely inhibited aberrant astrocyte secretion of inflammatory mediators (Rizzo et al. 2019a). In contrast, neutralization of additional monocyte-derived factors such as TNF-α, MCP-1 and IL-6 had minimal to no effect on astrocyte responses, identifying monocyte-derived IL-1ß as a key mediator of astrocyte secretion of inflammatory factors (Rizzo et al. 2019a). While inflammatory mediators such as IL-6 serve necessary homeostatic functions in the CNS, chronic elevation of astrocyte-derived inflammatory mediators, such as IL-6, can contribute to neurotoxic and even neurodegenerative outcomes (Kummer et al. 2021; Pons-Espinal et al. 2024; Vadodaria et al. 2021). Collectively, these findings emphasize the importance of CD16^+^ monocyte-astrocyte interactions in sustaining inflammatory signaling in the CNS, suggesting that modulation of monocyte activation may represent a promising target to limit HIV-associated neuroinflammation.

Studies report that 27–78% of individuals living with HIV actively use cannabis, whereas cannabis use among the general U.S. population is estimated to be 15% (Furler et al. 2004; Hahn et al. 2024; Health 2024). The reasons for cannabis use in the HIV community are diverse, ranging from symptom management, such as alleviating pain, anxiety, or appetite loss, to recreational use (Boehnke et al. 2019; Costiniuk et al. 2019; Haney et al. 2007; Harris et al. 2014). Furthermore, cannabis use among HIV individuals has been associated with decreased cerebrospinal fluid (CSF) levels of pro-inflammatory chemokines, monocyte chemoattractant protein-1 (MCP-1), and interferon-gamma-inducible protein 10 (IP-10), compared to non-cannabis-using HIV individuals (Watson et al. 2021). Interestingly, cannabis use has also been reported to alleviate pain and inflammation associated with other chronic conditions, including rheumatoid arthritis, fibromyalgia, and multiple sclerosis (Hershkovich et al. 2023; Paland et al. 2023; Yadav et al. 2014).

Previously published work in the literature has recognized the immune-modulating properties of Δ^9^-tetrahydrocannabinol (THC) and cannabidiol (CBD), which are the two major cannabinoids present in *Cannabis sativa* (Rizzo et al. 2019b; Sermet et al. 2021, 2025). Furthermore, THC and CBD have been reported as potential therapeutics for mitigating inflammation in animal models of rheumatoid arthritis (Arévalo-Martín et al. 2003; Baker et al. 2000; Malfait et al. 2000b; Smith et al. 1998). Previous studies from our laboratory have shown that THC suppresses inflammatory IL-6 and MCP-1 secretion from a human coculture model containing primary human pan monocytes and fetal astrocytes (Rizzo et al. 2019b). Additionally, THC and CBD suppress monocyte secretion of inflammatory cytokines, including IL-1ß and IL-6 (Sermet et al. 2021, 2025). Notably, IL-1ß release was potently inhibited by cannabinoid treatment through inhibition of its post-translational maturation, as evidenced by suppressed caspase-1 activity and canonical inflammasome formation (Sermet et al. 2025). While both cannabinoids suppress inflammatory cytokine release by monocytes, THC was more efficacious, highlighting the differences in intracellular signaling by THC and CBD. This can further be important for distinguishing the therapeutic potential of individual cannabinoids. Despite structural similarity and overlapping immunomodulatory effects, THC and CBD differ in their psychotropic activity: THC produces the characteristic “high” associated with cannabis by binding to cannabinoid receptor (CBR)1, whereas CBD has little to no affinity for CBR1, and does not induce psychotropic effects (Kaplan et al. 2008; Pertwee 2008). However, it is noteworthy that both THC and CBD have also been reported to interact with additional molecular targets beyond the CBRs, such as peroxisome proliferator-activated receptor γ (PPAR γ), transient receptor potential vanilloid (TRPV) receptors, and G-protein receptor 55 (GPR55), which may also contribute to their immunomodulatory effects (Adams et al. 1940; Devane et al. 1988; 656 Lauckner et al. 2008; Munro et al. 1993; O'Sullivan et al. 2005; Vara et al. 2013; Zhang et al. 2022). This distinction emphasizes the importance of studying both compounds because while THC demonstrates stronger immunosuppressive effects, CBD may offer anti-inflammatory benefits without the undesired psychotropic properties.

Although the anti-inflammatory activity of THC and CBD have been widely reported, the existing literature relies heavily on rodent models of rheumatoid arthritis, osteoarthritis, and multiple sclerosis (Al-Ghezi et al. 2019; Aswad et al. 2025; Furgiuele et al. 2021; Kozela et al. 2011; Malfait et al. 2000a). However, inflammatory responses in mice have been found to poorly mimic human inflammatory diseases (Seok et al. 2013). While animal models can provide valuable insights, human-based models, especially those using primary leukocytes, are often underappreciated, although necessary to confirm findings in other animal species. While CNS infiltrating CD16^+^ monocytes have been associated with neuroinflammation in HAND, to our knowledge, the inflammatory interactions between primary human CD16^+^ monocytes and brain resident cells have not yet been characterized. As astrocytes are an important regulator of CNS inflammatory signaling, elucidating how infiltrating CD16^+^ monocytes affect astrocyte inflammatory responses may provide insight as to how peripheral immune activation propagates neuroinflammation in HAND. Furthermore, this current study investigated for the first time whether cannabinoids, such as THC and CBD, can suppress CD16^+^ monocyte-astrocyte inflammatory responses, including inflammatory cytokine secretion and monocyte chemotaxis, utilizing primary human cells. We hypothesized that THC and CBD suppress CD16^+^ monocyte-induced astrocyte secretion of inflammatory mediators and monocyte recruitment via chemotaxis in the context of HIV. To test this hypothesis, we evaluated the effects of THC and CBD on inflammatory IL-6, MCP-1, and IL-8 production from primary human CD16^+^ monocyte-astrocyte cocultures, as well as their impact on the chemotactic capacity of monocytes isolated from HIV- and HIV+ individuals. Collectively, this work defines how THC and CBD regulate CD16^+^ monocyte-astrocyte inflammatory crosstalk and downstream monocyte chemotaxis in the context of neuroinflammation and HIV infection. By elucidating the interactions between CD16^+^ monocytes and astrocytes, this investigation builds upon the foundation to develop more complex in vitro systems for future studies incorporating additional cellular contributors of HAND, such as microglia, neurons, and CD8^+^ T cells.

## Methods

### Reagents and Chemicals

Roswell Park Memorial Institute Medium (RPMI) 1640 was purchased from Gibco™ by Life Technologies (Carlsbad, CA). Lyophilized CBD (CAS#: 13956-29-1) and THC suspended in 100% ethanol (CAS#: 1972-08-3) were obtained from the National Institute on Drug Abuse Drug Repository (Bethesda, MD). Lyophilized JWH-015 (CAS: 155471-08-2) was purchased from Cayman Chemical (Ann Arbor, MI). The following antibodies were purchased from BioLegend (San Diego, CA): anti-CD45-Pacific Blue (clone: H130), anti-CD14-PeCy7 (clone: 63D3), anti-CD16-PE (clone: 3G8), anti-CD56-PerCP (clone: HCD56), anti-CD57 PerCP/Cy5.5 (clone: HNK-1), anti-CD11b (activated)-APC (clone: CBRM1/5), anti-CD11a/CD18-PE (clone: m24). LIVE/DEAD Fixable Aqua Dead Cell Stain was purchased from ThermoFisher Scientific (Ann Arbor, MI). TLR agonists imiquimod (R837) and HIV-1 L-derived ssRNA40 were purchased from InvivoGen (San Diego, CA). CellMask™ Green Actin Tracking Stain was purchased from ThermoFisher Scientific (Waltham, MA).

### HIV Subject Recruitment

The HIV+ cohort in this study was recruited for blood draw under the Institutional Review Board (IRB) protocol (MSU Study ID: STUDY00006315) by Dr. Peter Gulick and Beth Leipprandt. HIV+ donors are enrolled in the Mid-Michigan HIV consortium. All HIV+ subjects in this current study are actively on anti-retroviral therapy (ART) and have undetectable viral loads. Demographic and immunological information on the HIV+ cohort is presented in Table 1.

**Table 1:** HIV+ subject demographic and immunological data. HIV: Human immunodeficiency virus; ART: Anti-retroviral therapy. Age, CD4 T cell count, CD4/CD8 ratio, and time infected with HIV are expressed as mean (standard deviation). Other drug use includes cocaine, methamphetamine, and heroin. Unequal N values in HIV+ (N) indicate missing patient information

Patient Information	HIV+subjects	HIV+subjects (N)
N	6	6
Age (years)	59.5 (13.2)	6
Sex	6 males	6
Race	2 Black, 4 White	6
Ethnicity	6 non-Hispanic	6
CD4 T-cell Count (cells/μL)	498.7 (171.83)	5
CD4/CD8 Ratio	0.94 (0.43)	5
Time Infected with HIV(years)	24 (11.7)	6
% on ART	100	6
% with Undetectable Viral Load(<200 copies/mL)	100	6
Cigarette Smoking (%)	16.7	6
Alcohol Use (%)	33.3	6
Other Drug Use (%)	0	6

### Monocyte Purification

Peripheral blood mononuclear cells (PBMC) were isolated from either enriched leukocyte packs (HIV-) or whole blood (HIV+). Enriched leukocyte packs were purchased from Gulf Coast Regional Laboratories and tested negative for HIV, hepatitis B virus, hepatitis C virus, and human T-cell lymphotropic virus. Leukocyte packs were shipped at 4 °C overnight. All other demographic and immunological information for HIV- donors were not disclosed. PBMCs were isolated by density gradient centrifugation using Ficoll-Paque PLUS (GE Healthcare Life Sciences). Pan monocytes were isolated from PBMCs by negative magnetic selection utilizing the Human Pan Monocyte Isolation Kit per the manufacturer (Miltenyi Biotec, Begisch Gladbach, Germany). For the isolation of CD16^+^ monocytes, pan monocytes were first stained with anti-CD16 (PE) for 15 min on ice, followed by positive magnetic selection using MojoSort™ Human anti-PE Nanobeads (Biolegend), according to the manufacturer’s instructions. CD16^+^ monocytes were subsequently collected for coculture experiments. Immediately following isolations, monocyte purities were assessed by flow cytometry as indicated in the Flow Cytometry Section. The average purity across all 48 monocyte donors included in these studies was 95.8% ± 2.76% (S.D.). Furthermore, donors used in coculture experiments had an average purity of 97.4% ± 1.37% (S.D.) (*n* = 24), with the lowest observed purity being 94%. Donors included in migration assay experiments had an average purity of 94.4% ± 3.2% (S.D.) (*n* = 14), with the lowest purity observed at 87%. All donors included in flow cytometry experiments had an average purity of 93.9% ± 2.4% (S.D.) (*n* = 8), with the lowest observed purity of 90.3%. Leukocytes were identified by cellular expression of CD45 (leukocyte common antigen). CD16^+^ (CD14^+^16^+^ and CD14^−^16^+^) and pan monocytes were further identified based on cell surface expression of CD14 and CD16, and lack of CD56 and CD57 (Natural Killer cell markers).

### Primary Human Astrocyte Cell Culture

Primary normal human astrocytes (NHA) isolated from cerebral cortex were purchased from Sciencell Research Laboratories (Carlsbad, CA) and cultured according to the manufacturer’s protocol in complete astrocyte medium supplemented with growth supplements (ScienCell Research Laboratories). Media was changed every other day for subculturing and seeding of culture plates. A total of three distinct NHA donors were utilized across the following experiments (donor sex is unknown). Of this, two distinct donors were utilized for coculture experiments, and supernatants were collected from one distinct donor for migration assay experiments.

### CD16^+^ Monocyte-Astrocyte Cocultures

NHAs were seeded in 24-well plates in complete astrocyte media 3–4 days prior to coculture at a cell density that would estimate to be 2.4 × 10^5^ cells/mL on the day of coculture. CD16^+^ monocytes were isolated on the day of coculture and seeded into wells containing astrocytes at 1.2 × 10^4^ cells/mL to establish a previously determined monocyte to astrocyte ratio of 1:20 (Rizzo et al. 2019a, b). Monocyte and astrocyte monoculture control wells were also seeded in parallel at the same density as coculture wells in 24-well plates. Culture wells were pre-treated with cannabinoids THC or CBD (0.5, 1, 5, and 10µM) for 30 min at 37 °C prior to activation with either R837 (TLR7 agonist- 10 µg/mL) or ssRNA40 (TLR8 agonist-0.5 µg/mL). There were a total of three experimental groups within coculture experiments: untreated (UT), TLR activation only, and TLR-activated cocultures treated with either Vh or THC/CBD (0.5, 1, 5, 10µM per cannabinoid). The vehicle concentration for the Vh control and cannabinoid treatments was 0.03% ethanol. Cells were incubated at 37 °C and 5% CO_2_ for 22 h in accordance with previous monocyte culture studies conducted in our laboratory (Rizzo et al. 2019a, b; Sermet et al. 2025). This timepoint was selected based on established monocyte cytokine kinetics, wherein peak cytokine production occurs approximately within 15-hours post-activation, allowing sufficient time for downstream monocyte-mediated effects on astrocytes to manifest and be assessed at the 22-hour endpoint. Following incubation, cells were stained with LIVE/DEAD Fixable Aqua, and viability was assessed by flow cytometry as indicated in the Flow Cytometry Section. Cell viability following cannabinoid treatments was determined to be ≥ 90% (data not shown).

### Monocyte and Astrocyte Monocultures

Primary CD16^+^ monocyte and NHA monoculture control wells were seeded in 24-well plates alongside coculture wells to determine individual cell contributions in cocultures. Cells were seeded at the same density as what was described in the CD16^+^monocyte-Astrocyte Coculture Section for coculture wells. CD16^+^ monocyte monoculture wells were pre-treated with THC or CBD (0.5, 1, 5, 10µM) for 30 min at 37 °C prior to activation with either R837 (TLR7 agonist-10 µg/mL) or ssRNA40 (TLR8 agonist-0.5 µg/mL). There was a total of three experimental groups within the monocyte monoculture wells: untreated (UT), TLR activation only, and TLR-activated monocultures treated with either Vh or THC/CBD (0.5, 1, 5, 10µM per cannabinoid). NHA monoculture wells were pre-treated with THC or CBD (10µM) for 30 min, then stimulated with recombinant IL-1ß (20pg/mL). Some NHA monocultures were treated with only R837 (10 µg/mL) or ssRNA40 (0.5 µg/mL) to serve as a control. There were a total of four experimental groups within the NHA monoculture control wells: untreated (UT), TLR activation only, recombinant IL-1ß only, and recombinant IL-1ß treated with either Vh or THC/CBD (10µM per cannabinoid). The vehicle concentration for the Vh control and cannabinoid treatments was 0.03% ethanol. All monocultures were incubated at 37 °C and 5% CO_2_ for 22 h.

### Supernatant Measurements of Cytokines/Chemokines

Following 22 h of incubation, coculture, CD16^+^ monocyte monoculture, and NHA monoculture plates were centrifuged at 330 g for 5 min, then supernatants were transferred into new 24-well culture plates and stored at −80 °C. Supernatants were quantified for secreted IL-6, IL-8, MCP-1, and IL-1ß utilizing ELISAmax™ assays (Biolegend). Colorimetric signals were immediately recorded on a BioTek Synergy HTX multimode reader (Agilent Technologies, Santa Clara, CA).

### Flow Cytometry

Staining buffer (PBS, 1% bovine serum albumin, and 0.1% NaN_3_) was used to wash cells between staining and fixation steps. Prior to staining with the specified antibodies, cells were incubated for 20 min at 4 °C with staining buffer containing 20% human AB serum to block Fc receptors. 4% paraformaldehyde fixation buffer (BD Biosciences, Franklin Lakes, NJ) was used to fix cells. Stained samples were analyzed on a Cytek Northern Lights (Cytek Biosciences, Fremont, CA), and data analysis was performed using FlowJo software (Version 10.10.0, Treestar Software, Ashland, OR).

### Astrocyte Conditioned Media Collection for Chemotaxis Assay

Prior to chemotaxis experiments, conditioned media was collected from NHA cultures. NHA were cultured as indicated in the Primary Human Astrocyte Cell Culture Section and seeded into 24-well plates as indicated in the CD16^+^Monocyte-Astrocyte Cocultures Section. On treatment day, NHA were either untreated or treated with 300pg/mL recombinant IL-1ß in incomplete serum-free astrocyte medium (ScienCell Research Laboratories). Cells were incubated at 37 °C and 5% CO_2_. 22 h following treatment, NHA culture plates were centrifuged at 330 g for 5 min, and the supernatants were saved, aliquoted, then stored at −80 °C for subsequent chemotaxis assays. NHA conditioned media aliquots were never thawed more than once.

### Chemotaxis Assay

Pan monocytes from HIV- and HIV+ donors were isolated from PBMC as indicated in the Monocyte Purification Section and used in the following chemotaxis experiments. Monocytes were seeded at 2.5 × 10^5^ cells/mL into top chambers (reservoir inserts) of Incucyte Clearview 96-well Microplate for Chemotaxis (Sartorius AG, Gottingen, Germany) in incomplete serum-free astrocyte media (ScienCell Research Laboratories) per the manufacturer’s instructions. Incomplete NHA media, Vh, or THC/CBD (0.5, 1, 5, 10µM per cannabinoid) was added to the top chambers for monocyte pre-treatment. Monocytes were allowed to settle in reservoir inserts at ambient temperature for 1 h. Following 1 h pre-treatment, 200uL fresh incomplete astrocyte media, fresh incomplete astrocyte media supplemented with recombinant IL-1ß (300pg/mL) or recombinant MCP-1 (2 µg/mL), or astrocyte conditioned media (pre-warmed to ambient temperature) was added to bottom chambers (reservoir plate). The chemotaxis plate system was immediately incubated at 37 °C and 5% CO_2_ for 2 h. After 2-hour incubation, reservoir inserts (top chambers) were removed, and only the reservoir plate was centrifuged at 330 g for 5 minutes. The bottom of the reservoir plate was imaged using the Incucyte S3 Live-Cell Analysis System (Incucyte-2023 A-Rev2 Software, Sartorius) to quantify the number of migrated monocytes.

### Monocyte Integrin Quantification

Pan monocytes from HIV- subjects were isolated as indicated in the Monocyte Purification Section and seeded in 96-well plates at a cell density of 8.33 × 10^5^ cells/mL. Monocytes were treated with THC or CBD (0.5, 1, 5, 10µM) for 1 h at ambient temperature in incomplete serum-free astrocyte media (ScienCell Research Laboratories) to mimic the cannabinoid pre-treatments conducted in the chemotaxis protocol. Following the 1-hour treatment, supernatants were removed, and cells were surface-stained with antibodies directed against the high-affinity active conformational state of integrins LFA-1 (CD11a) and MAC-1 (CD11b), as indicated in the Flow Cytometry Section.

### F-Actin Determination

Pan monocytes from HIV- donors were isolated as indicated in the Monocyte Purification Section and seeded in 96-well plates at a cell density of 8.33 × 10^5^ cells/mL. Monocytes were treated with THC or CBD (0.5, 1, 5, 10µM) for 1 h at ambient temperature in incomplete serum-free astrocyte media (ScienCell Research Laboratories) to mimic the cannabinoid pre-treatments conducted in the chemotaxis protocol. Following the 1-hour treatment, supernatants were removed and PBS containing CellMask Green Actin Tracking Stain (diluted 1:1000) was added to each well. Cells were stained for 30 min per the manufacturer’s instructions. Cells were washed three times, then surface-stained with antibodies directed against CD14 and CD16 as indicated in the Flow Cytometry Section.

### Statistical Analysis

Statistical analysis was performed using Prism 10.1.0 (GraphPad, San Diego, CA). Non-stimulated untreated (UT), TLR stimulation only, and vehicle (Vh) groups from cocultures and monocyte monocultures were analyzed using a repeated-measures one-way ANOVA, followed by a Tukey’s multiple-comparisons post hoc test to compare means between treatment groups. For assessment of astrocyte monocultures, non-stimulated untreated (UT), TLR stimulation only, recombinant IL-1ß, and recombinant IL-1ß in combination with THC or CBD were analyzed by repeated-measures one-way ANOVA, followed by Tukey’s multiple comparisons post hoc test. Due to variation in cytokine production among human donors, the measurements from cannabinoid-treated groups were normalized to the corresponding Vh control group for each donor. Cannabinoid-mediated effects on cytokine secretion, integrin expression, and actin polymerization were assessed using a repeated measures one-way ANOVA, followed by a Dunnett’s multiple comparisons post hoc test to compare each cannabinoid-treated group with the Vh control. HIV- and HIV+ monocyte chemotaxis towards fresh incomplete astrocyte media, recombinant IL-1ß, recombinant MCP-1, untreated astrocyte conditioned media, IL-1ß treated astrocyte conditioned media, and IL-1ß treated astrocyte conditioned media plus Vh pre-treatment were analyzed by repeated measures one-way ANOVA, followed by Dunnett’s multiple comparisons post hoc test to compare the means to the fresh incomplete astrocyte media control. To account for variation in chemotaxis capacity in human donors, the number of migrated cells in response to astrocyte-conditioned media was normalized to each donor’s respective fresh incomplete astrocyte control or Vh pre-treated control. A two-way ANOVA with Tukey’s post hoc test was conducted to assess differences in normalized chemotaxis toward astrocyte conditioned media from the fresh incomplete astrocyte media control within each donor group and between HIV- and HIV+ donor groups. THC or CBD pre-treatment effects on chemotaxis were assessed by two-way ANOVA with Sidak’s post hoc test to compare cannabinoid-treated effects from Vh control within and between donor groups.

## Results

### THC and CBD Treatments Suppress Astrocyte IL-6, MCP-1, and IL-8 Secretion in TLR7-Stimulated Cocultures

**Fig. 1:**
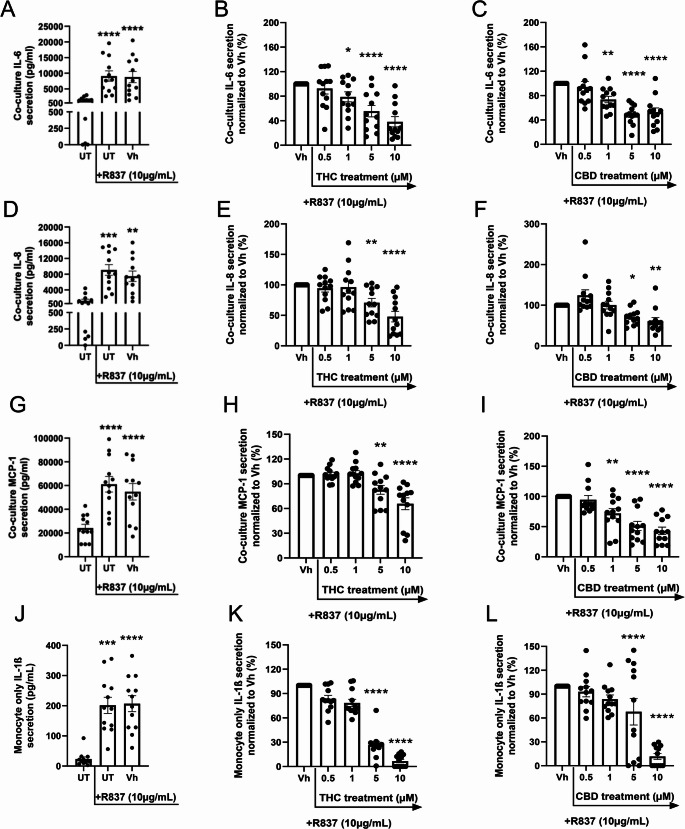
**THC and CBD suppress CD16**^**+**^** monocyte-initiated IL-6, IL-8, and MCP-1 secretion from astrocytes in TLR7-activated human cocultures. ** (**A**-**I**) Primary human CD16^+^ monocytes were cocultured with primary human astrocytes in a 1:20 ratio. (**J**-**L**) Monocytes were monocultured in parallel at the same density as conducted in coculture conditions. (**A**, **D**, **G**, **J**) Cocultures and monoculture were either untreated (UT), activated with R837 (10 µg/mL), or activated with R837 in combination with vehicle (0.03% ethanol; Vh). Following a 22 hour incubation, supernatant IL-6, IL-8, MCP-1, and IL-1ß were quantified by ELISAmax™. The graphs represent the unnormalized concentrations of each mediator. Asterisks denote statistically significant differences from the unstimulated control in each group (**p < 0.01, ***p < 0.001, ****p < 0.0001) as determined by repeated measures one-way ANOVA with Tukey's post hoc test. (**B**, **C**, **E**, **F**, **H**, **I**, **K**, **L**) Cocultures and monocultures were activated with R837 and treated with Vh, THC, or CBD (0.5, 1, 5, 10 µM). Cells were cultured for 22 hours, then supernatant IL-6, IL-8, MCP-1, and IL-1ß were quantified by ELISAmax™. After cytokine quantification, values were normalized to the Vh-treated control and expressed as a percentage. Asterisks denote statistically significant differences from the Vh control in each group (*p < 0.05, **p < 0.01, ****p < 0.0001) as determined by repeated measures one-way ANOVA with Dunnett's post hoc test. All graphs show the mean ± S.E.M. (A-L). (n = 12 monocyte donors, n = 2 astrocyte donors)

We previously established that monocyte-derived IL-1ß is an inducing factor for astrocyte secretion of inflammatory mediators IL-6 and MCP-1 in a primary human pan monocyte and human fetal astrocyte coculture, and that THC suppresses this response (Rizzo et al. 2019a, b). However, because CD16^+^ monocytes, a minor population within the pan monocyte pool, have been implicated in HAND, we sought to further understand how cocultures containing CD16^+^ monocytes and astrocytes respond to cannabinoid treatment. Here, CD16^+^ monocytes and astrocytes were cocultured at a previously established ratio of 1:20 (Rizzo et al. 2019a, b), pretreated with THC or CBD (0, 0.5, 1, 5, 10µM), and subsequently stimulated with TLR7 agonist R837 (10 µg/mL) for 22 h. R837 significantly induced IL-6, IL-8, and MCP-1 secretion from cocultures compared to background levels (Fig. 1A, D, and G). THC treatment significantly suppressed IL-6 secretion at 1, 5, and 10µM (Fig. 1B), whereas IL-8 and MCP-1 were significantly suppressed at 5 and 10µM THC (Fig. 1E and H). Similarly, CBD treatment significantly impaired secretion of IL-6 and MCP-1 at 1, 5, and 10 µM (Fig. 1C and I), and IL-8 at 5 and 10 µM (Fig. 1F) in the coculture.

Given the role of monocyte-derived IL-1ß in inducing inflammatory mediator release in this coculture system, IL-1ß secretion was quantified in CD16^+^ monocyte monocultures seeded at the same density as used under coculture conditions. Monocyte IL-1ß release was significantly induced by R837 stimulation compared to unstimulated controls (Fig. 1J). Further, both THC and CBD treatment significantly suppressed monocyte IL-1ß secretion at 5 and 10µM (Fig. 1K and L). IL-1ß production by astrocytes in monoculture was also determined; however, it was below the level of quantification (not shown).

**Fig. 2:**
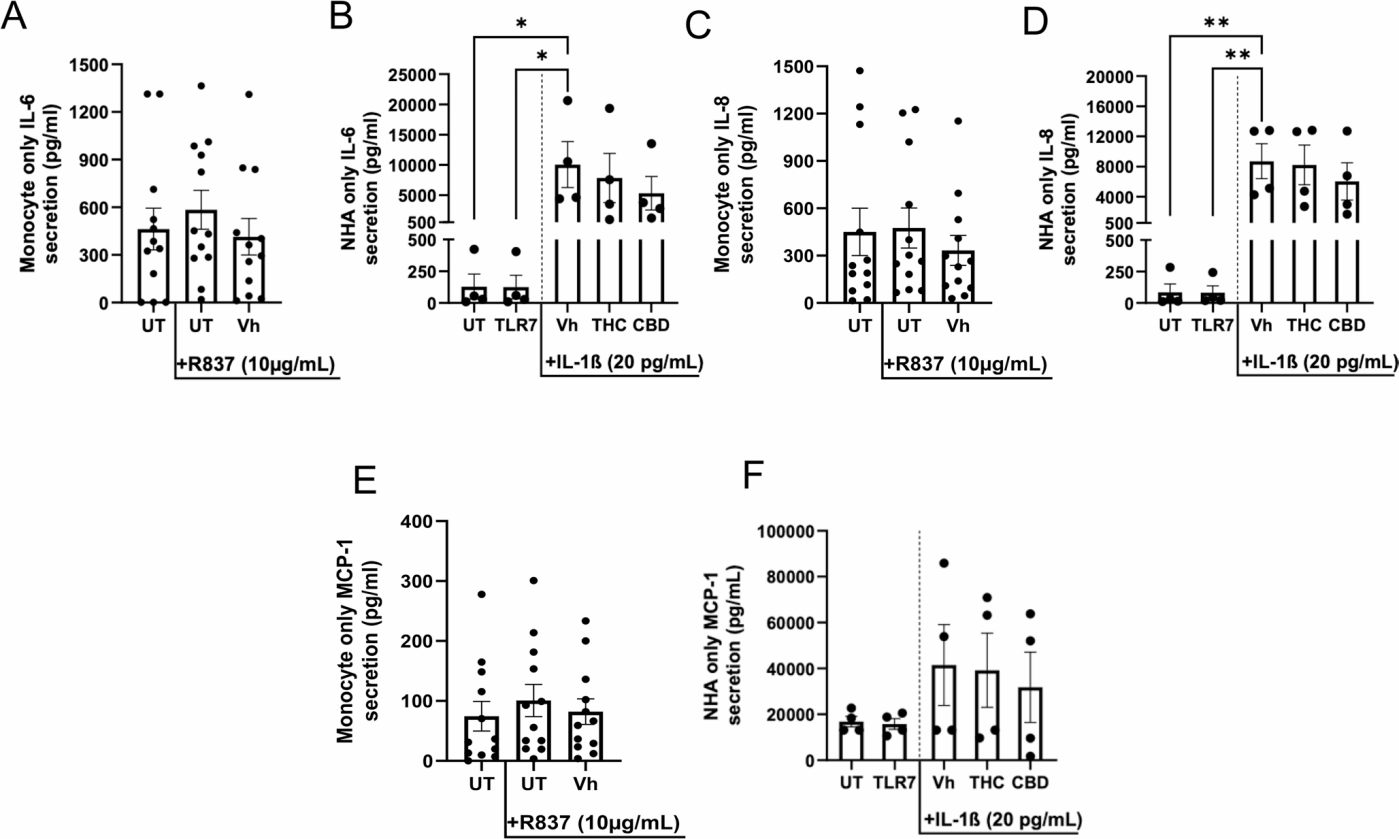
**Astrocytes are the major contributors of inflammatory factors in TLR7-activated cocultures. ** (**A**,**C**, **E**) Primary human CD16^+^ monocultures and (**B**, **D**, **F**) primary human astrocyte monocultures were cultured in parallel at the same density as conducted in coculture conditions. (**A**, **C**, **E**) Monocytes were either untreated, activated with R837 (10 µg/mL), or activated with R837 in combination with vehicle (0.03% ethanol; Vh). (**B**, **D**, **F**) Astrocytes were either untreated, treated with R837 (10 µg/mL), or activated with recombinant IL-1ß in combination with Vh, THC or CBD (10 µM). (**A**-**F**) After a 22 hr incubation, supernatant IL-6, IL-8, and MCP-1 were quantified by ELISAmax™. These graphs represent the unnormalized concentrations of each mediator. Asterisks denote statistically significant differences from the unstimulated control (*p < 0.05, **p < 0.01) as determined by repeated measures one-way ANOVA with Tukey's post hoc test. All graphs show the mean ± S.E.M. (**A**-**F**). (n = 12 monocyte donors, n = 2 astrocyte donors with 2 experimental replicates each)

To determine which cell type within the TLR7-stimulated coculture system was responsible for the release of inflammatory mediators, we next quantified supernatant release of IL-6, IL-8,and MCP-1 from CD16^+^ monocyte monocultures and astrocyte monocultures seeded at the same density as in coculture conditions. Although CD16^+^ monocyte monocultures released low levels of IL-6, IL-8, and MCP-1, R837 stimulation did not significantly induce production beyond background levels (Fig. 2 A, C, and E). While astrocyte monocultures produced little to no background levels of IL-6 and IL-8, and some MCP-1 release was observed, TLR7 treatment did not stimulate secretion (Fig. 2B, D, and F). By contrast, treatment of astrocytes with recombinant IL-1ß (20 pg/mL) significantly increased the release of IL-6 and IL-8 above background levels (Fig. 2B and D). MCP-1, although increased, was not significantly enhanced with recombinant IL-1ß treatment (Fig. 2 F). Furthermore, the capacity of the astrocytes to release IL-6, IL-8, and MCP-1 was 17.21-, 18.33-, and 415.07-fold higher than what is observed in monocyte monocultures, respectively, suggesting that the astrocytes were primarily responsible for inflammatory mediator release in monocyte-astrocyte cocultures.

Additionally, THC and CBD treatments did not significantly affect IL-6, IL-8, or MCP-1 secretion by astrocyte monocultures, suggesting that the cannabinoid effects observed under coculture conditions were unlikely to be due to direct effects on astrocytes (Fig. 2B, D, and F).

### THC and CBD Treatments Suppress Astrocyte IL-6, MCP-1, and IL-8 Secretion in TLR8-Stimulated Cocultures

**Fig. 3:**
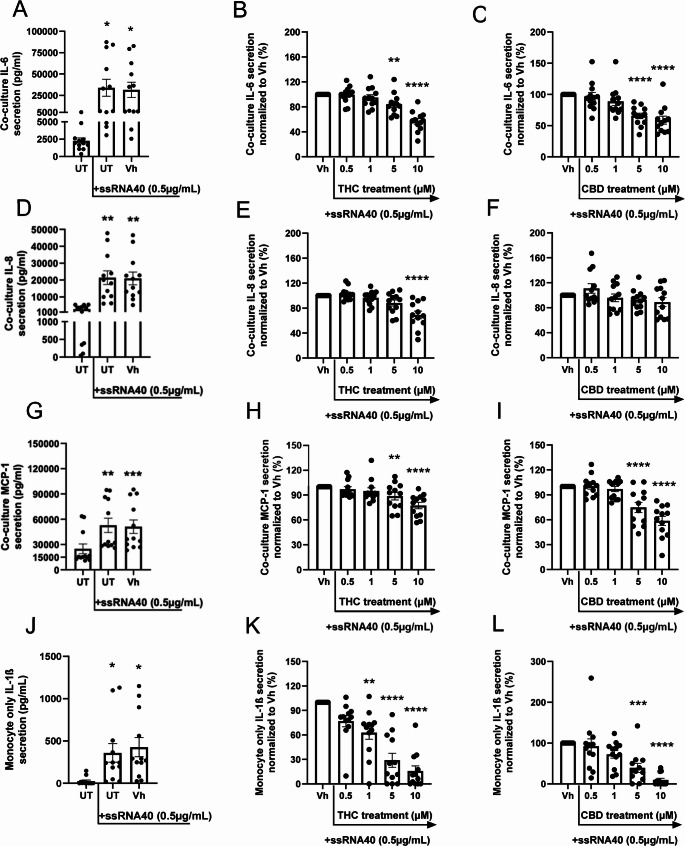
**THC and CBD inhibit CD16**^**+**^** monocyte-mediated IL-6, IL-8, and MCP-1 secretion from astrocytes in TLR8-activated human cocultures. ** (**A**-**I**) Primary human CD16^+^ monocytes were cocultured with primary human astrocytes in a 1:20 ratio. (**J**-**L**) Monocytes were monocultured in parallel at the same density as conducted in coculture conditions. (**A**, **D**, **G**, **J**) Cocultures and monoculture were either untreated (UT), activated with ssRNA40 (0.5 µg/mL), or activated with ssRNA40 in combination with vehicle (0.03% ethanol; Vh). Following a 22 hour incubation, supernatant IL-6, IL-8, MCP-1, and IL-1ß were quantified ELISAmax™. These graphs represent the unnormalized concentrations of each mediator. Asterisks denote statistically significant differences from the unstimulated control in each group (*p < 0.05, **p < 0.01, ***p< 0.001) as determined by repeated measures one-way ANOVA with Tukey's post hoc test. (**B**, **C**, **E**, **F**, **H**, **I**, **K**, **L**) Cocultures and monocultures were activated with ssRNA40 and treated with Vh, THC, or CBD (0.5, 1, 5, 10 µM). Cells were cultured for 22 hours, then supernatant IL-6, IL-8, MCP-1, and IL-1ß were quantified by ELISAmax™. After cytokine quantification, values were normalized to the Vh-treated control and expressed as a percentage. Asterisks denote statistically significant differences from the Vh control in each group (**p < 0.01, ***p < 0.001, ****p < 0.0001) as determined by repeated measures one-way ANOVA with Dunnett's post hoc test. All graphs show mean ± S.E.M. (A-L). (n = 12 monocyte donors, n = 2 astrocyte donors)

Monocytes express high levels of TLR8, in addition to TLR7, both of which can recognize and activate in the presence of HIV ssRNAs (Guo et al. 2014; Heil et al. 2004). Therefore, we also evaluated the effect of cannabinoid treatment under monocyte-astrocyte coculture conditions when activated through TLR8. Cocultures were seeded at a 1:20 CD16^+^ monocyte-astrocyte ratio, then pretreated with THC or CBD (0.5, 1, 5, 10µM), and subsequently stimulated with TLR8 agonist, ssRNA40 (0.5 µg/mL). TLR8 stimulation induced the release of IL-6, IL-8, and MCP-1 in the coculture (Fig. 3 A, D, and G). THC treatment suppressed IL-6 and MCP-1 secretion at 5 and 10µM (Fig. 3B and H), whereas IL-8 secretion was significantly suppressed but only at the 10µM concentration (Fig. 3E). Additionally, CBD treatment suppressed TLR8-induced release of IL-6 and MCP-1 at 5 and 10µM (Fig. 3 C and I), while IL-8 production was unaffected by CBD treatment (Fig. 3 F). CD16^+^ monocyte monoculture release of IL-1ß was also assessed, which indicated that ssRNA40 significantly enhanced IL-1ß release compared to background levels (Fig. 3 J). THC suppressed TLR8-induced monocyte IL-1ß secretion at 1, 5, and 10µM treatments, whereas CBD suppressed IL-1ß secretion at 5 and 10µM concentrations (Fig. 3 K and L).

**Fig. 4:**
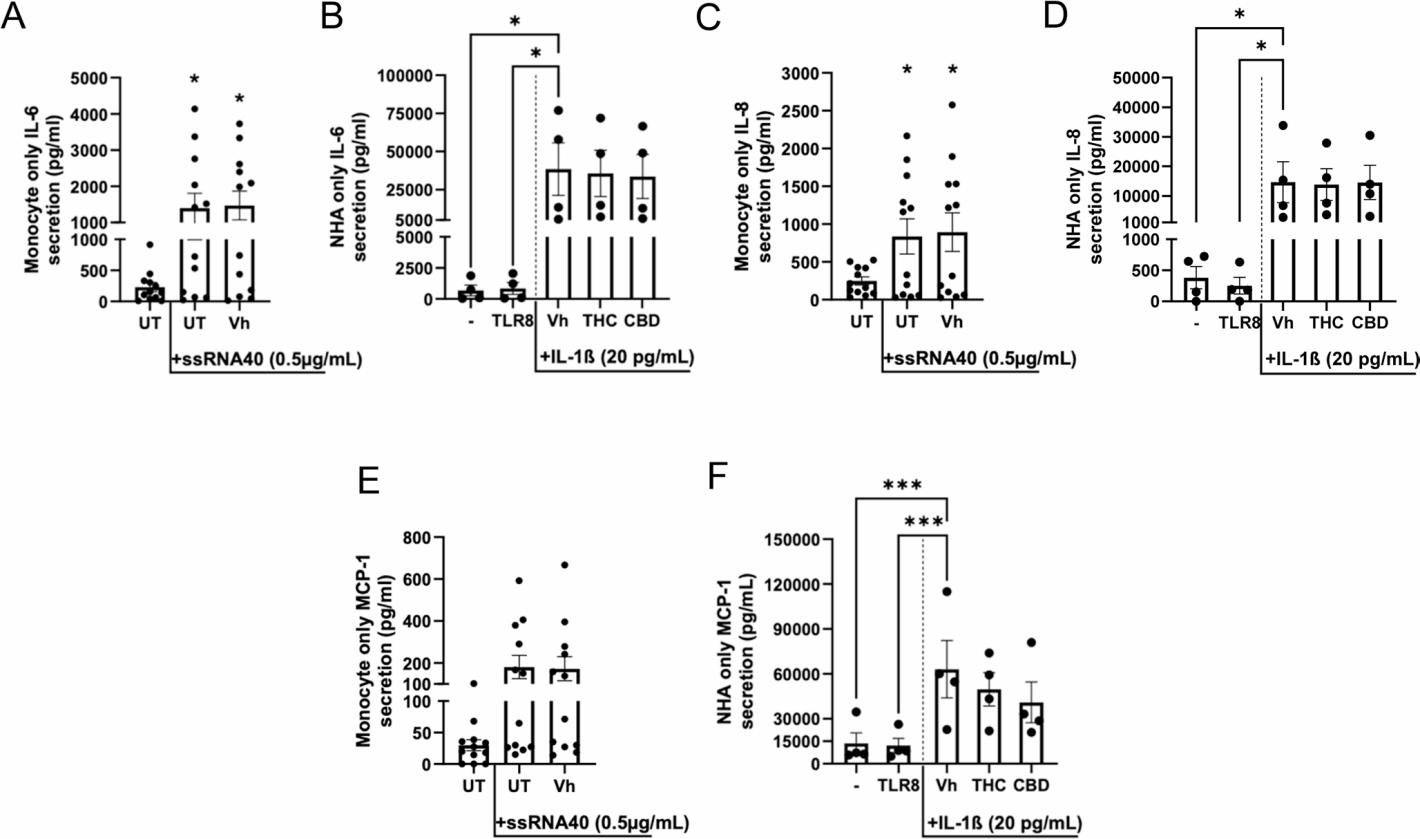
**Astrocytes are the major contributors of inflammatory factors in TLR8-activated cocultures. ** (**A**,**C**, **E**) Primary human CD16^+^ monocultures and (**B**, **D**, **F**) primary human astrocyte monocultures were cultured in parallel at the same density as conducted in coculture conditions. (**A**, **C**, **E**) Monocytes were either untreated, activated with ssRAN40 (0.5 µg/mL), or activated with ssRNA40 in combination with vehicle (0.03% ethanol; Vh). (**B**, **D**, **F**) Astrocytes were either untreated, treated with ssRNA40 (10 µg/mL), activated or activated with recombinant IL-1ß in combination with Vh, THC or CBD (10 µM). (**A**-**F**) After a 22 hr incubation, supernatant IL-6, IL-8, and MCP-1 were quantified by ELISAmax™. These graphs represent the unnormalized concentrations of each mediator. Asterisks denote statistically significant differences from the unstimulated control (*p < 0.05, **p < 0.01) as determined by repeated measures one-way ANOVA with Tukey's post hoc test. All graphs show the mean ± S.E.M. (A-F). (n = 12 monocyte donors, n = 2 astrocyte donors with 2 experimental replicates each)

To determine which cell type within the TLR8-stimulated coculture was responsible for the release of inflammatory mediators, we next quantified supernatant release of IL-6, IL-8, and MCP-1 from CD16^+^ monocyte monocultures and astrocyte monocultures seeded at the same density as used in coculture. ssRNA40 treatment significantly induced CD16^+^ monocyte monoculture release of IL-6 and IL-8 compared to background (Fig. 4 A and C), whereas MCP-1 was not significantly induced (Fig. 4E). Similar to R837, ssRNA40 treatment did not induce the production of inflammatory mediators by astrocytes; however, marked induction was observed when astrocyte monocultures were treated with recombinant IL-1ß (Fig. 4B, D, and F). Astrocyte monoculture production of IL-6, IL-8, and MCP-1 were 27.45-, 17.52-, and 348.75-fold higher than what was observed in monocyte monocultures, respectively, suggesting that the astrocytes were the major contributors to inflammatory mediators under coculture conditions. Furthermore, it appears that astrocyte release of inflammatory factors under coculture conditions was mediated by IL-1ß released from TLR8-stimulated CD16^+^ monocytes.

Additionally, astrocyte production of IL-6, IL-8, and MCP-1 was not affected by THC and CBD treatment (10µM), suggesting that cannabinoid effects observed under coculture conditions were not likely due to direct effects on the astrocytes (Fig. 4B, D, and F).

### Chemotaxis is Enhanced in Monocytes from HIV+ Individuals Compared to HIV- and is Significantly Suppressed by THC Treatment

**Fig. 5:**
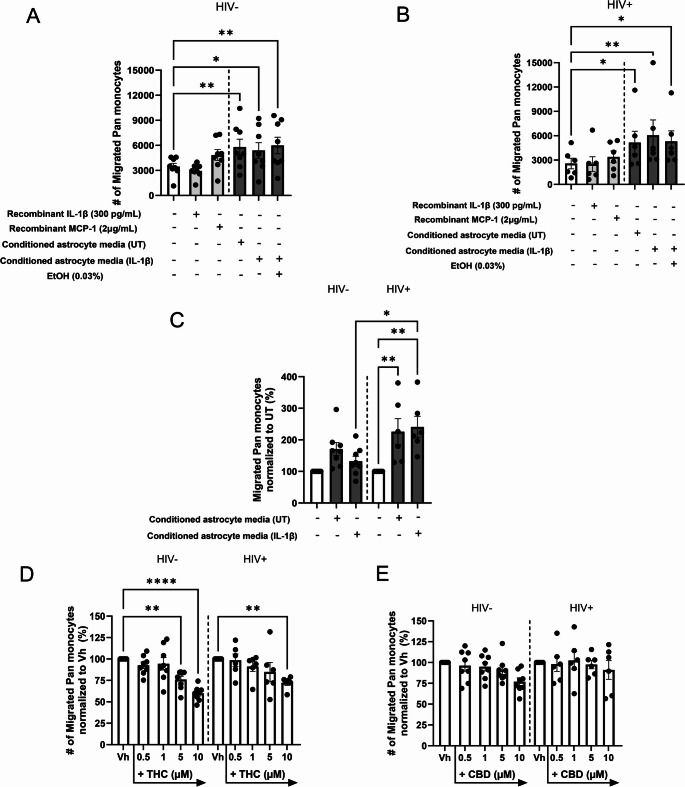
**Chemotaxis is enhanced in monocytes derived from HIV+ subjects and is inhibited by THC pre-treatment in HIV+ and HIV- monocytes.** (**A**-**E**) Pan monocytes isolated from HIV- (**A**, **C**, **D**, **E**) and HIV+ (**B**, **C**, **D**, **E**) individuals were seeded into the top chamber of chemotaxis plates in serum-free media (**A**-**C**) or serum-free media supplemented with Vehicle (0.03% ethanol; Vh), THC, or CBD (0.5, 1, 5, 10 µM) (**D**, **E**). After a 1 hr incubation to allow cells to settle, (**A**-**C**) fresh serum-free media supplemented with recombinant IL-1ß or MCP-1, or recombinant IL-1ß (300pg/mL) untreated/treated astrocyte conditioned media was added to the bottom chambers. (D, E) IL-1ß-treated astrocyte conditioned media was added to the bottom chambers. (**A**-**E**) Monocytes were incubated for 2 hours, then the number of migrated monocytes were quantified via Incucyte S3. (**A**, **B**) Asterisks denote statistical differences from the serum-free media control as determined by one-way ANOVA with Dunnett’s post hoc test. (**C**) Asterisks denote statistically significant differences between means within and between HIV- and HIV+ groups (*p < 0.05, **p < 0.01) as determined by two-way ANOVA with Tukey's post hoc test. (**D**, **E**) Asterisks denote statistically significant differences from the Vh treated control within each donor group or between HIV- and HIV+ groups (**p < 0.01, ****p < 0.0001) as determined by two-way ANOVA with Sidak's post hoc test. All graphs show the mean ± S.E.M. (**A**-**E**) (n = 8 HIV- donors, n = 6 HIV+ donors)

As peripheral monocyte infiltration into the CNS is implicated in HAND, we next evaluated the chemotactic capacity of peripheral pan monocytes obtained from HIV- and HIV+ individuals (Clay et al. 2007; Fischer-Smith et al. 2001). Monocyte chemotaxis was assessed in response to conditioned media from primary astrocytes treated with and without recombinant IL-1ß. While recombinant MCP-1 did not elicit chemotaxis, pan monocytes from both HIV- and HIV+ individuals exhibited significantly increased migration towards astrocyte-conditioned media compared with background levels observed in the serum-free media control (Fig. 5A and B). Notably, this chemotactic response was observed regardless of whether astrocytes were exposed to recombinant IL-1ß. To directly assess differences in chemotactic responses of monocytes from HIV- and HIV+ subjects towards astrocyte conditioned media groups, migration of monocytes toward astrocyte media was expressed relative to each donor’s serum-free media control, to account for donor-to-donor variability in baseline migration (Fig. 5C). When normalized, monocytes obtained from HIV+ subjects exhibited significantly increased chemotaxis towards astrocyte conditioned media, regardless of recombinant IL-1ß treatment. Astrocyte-conditioned media did not significantly induce monocyte migration compared to the serum-free media control from HIV- subjects, when normalized. Importantly, monocytes from HIV+ individuals demonstrated significantly enhanced chemotaxis towards IL-1ß treated conditioned media compared to monocytes from HIV- individuals (Fig. 5C).

Finally, the effects of THC and CBD pre-treatment on monocyte chemotaxis were also assessed (Fig. 5D and E). Pre-treatment of HIV- monocytes with THC significantly suppressed their migratory capacity in a concentration-dependent manner, wherein inhibition of the response was observed at 5 and 10µM THC treatment (Fig. 5D). Although a concentration-dependent suppression of HIV+ monocyte chemotaxis was also observed, only the 10µM THC treatment significantly suppressed the response. There was no significant difference in the THC-mediated suppression of migration between monocytes obtained from HIV- and HIV+ subjects (Fig. 5D). Interestingly, CBD pre-treatment did not significantly affect monocyte migration in either donor group, although did appear to trend toward a suppression at 10µM CBD in the HIV- donor group (Fig. 5E).

### THC Pre-Treatment Suppresses Monocyte Expression of the High-Affinity Conformation State of Integrins LFA-1 and MAC-1

**Fig. 6:**
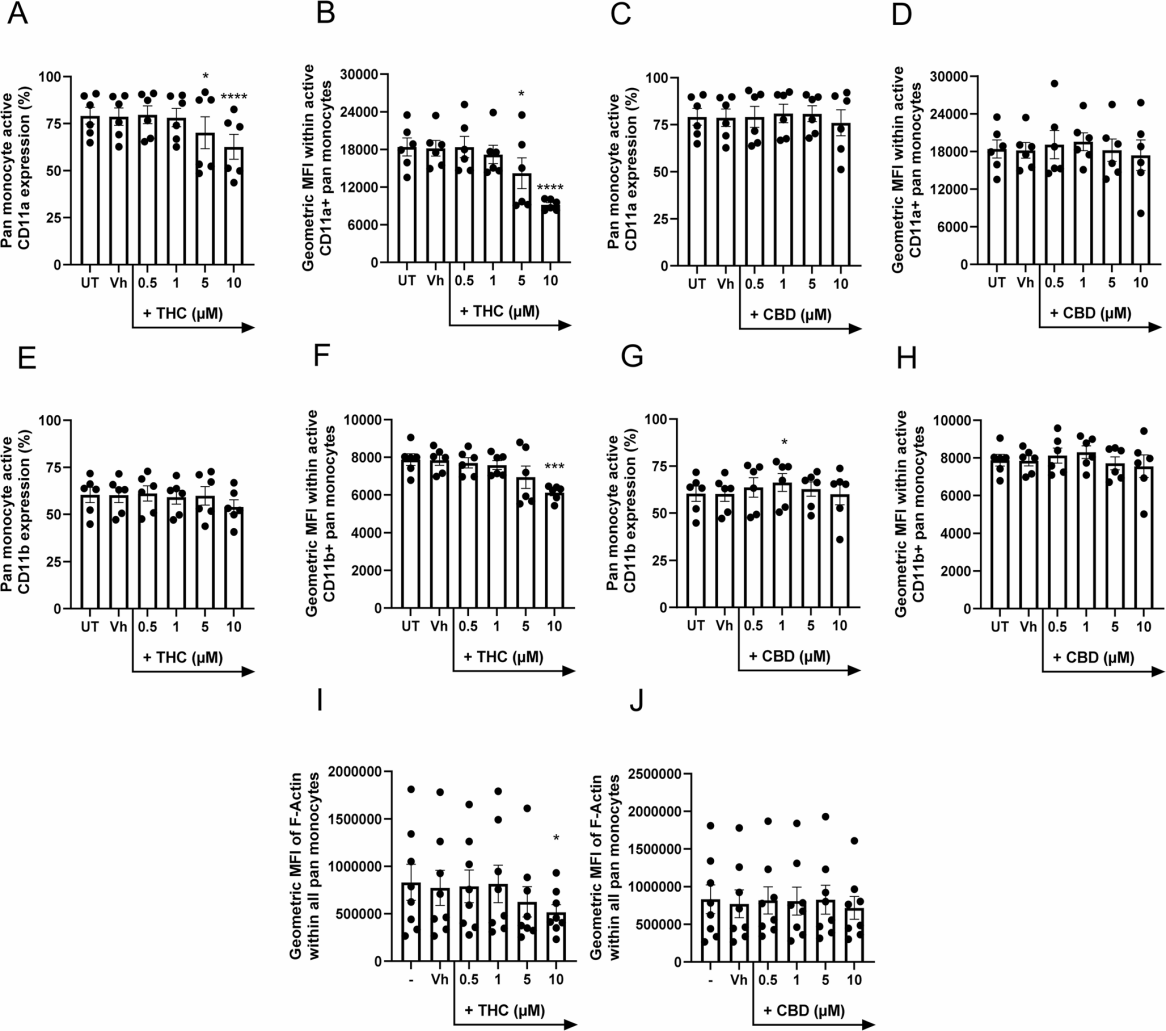
**Short-term THC treatment inhibits monocyte expression of high-affinity LFA-1 and MAC-1 integrins, as well as actin polymerization. ** (**A**-**J**) Pan monocytes were either untreated, treated with Vehicle (0.03% ethanol; Vh), THC, or CBD (0.5, 1, 5, 10 µM) for 1 hour at ambient temperature. Following the incubation, cells were immediately surface-stained utilizing antibodies directed against active conformations of integrins LFA-1 (CD11a), MAC-1 (CD11b), or F-Actin, then assessed by flow cytometry. (**A**, **C**, **E**, **G**) Represent the percent of monocytes expressing the active conformations of LFA-1 (**A**, **C**) or MAC-1 (**E**, **G)**. (**B**, **D**, **F**, **H**) Represent the geometric MFI within monocytes expressing the active conformations of LFA-1 (**B**, **D**) or MAC-1 (**F**, **H**). (**I**, **J**) Represent the geometric MFI of F-actin within all pan monocytes. (**A**-**J)** Asterisks denote statistically significant differences from the Vh control in each group (*p < 0.05, ***p < 0.001, ****p < 0.0001) as determined by repeated measures one-way ANOVA with Dunnett's post hoc test. All graphs show the mean ± S.E.M. (A-H) (n = 6), (I and J) (n = 8)

Since our results indicated that THC treatment suppresses monocyte migratory capacity, we next examined whether THC pre-treatment alters the expression of LFA-1 and MAC-1, surface integrins critical for monocyte chemotaxis (Sumagin et al. 2010). Integrins are dynamic receptors that cycle between a low-affinity/inactive state and a high-affinity/active state (Li et al. 2017; Takagi et al. 2002). To evaluate how short-term THC and CBD treatments influence the conformational states of LFA-1 and MAC-1, we utilized antibodies specific for the high-affinity conformations (Fig. 6A-H). Treatment with THC at 5 and 10µM significantly reduced both the percentage of monocytes expressing the high-affinity conformation of LFA-1 (Fig. 6 A) and the geometric mean fluorescence intensity (gMFI) after just 1 h of exposure (Fig. 6B). Additionally, while THC did not alter the percentage of monocytes expressing high-affinity MAC-1 (Fig. 6E), it significantly decreased MAC-1 gMFI at 10µM (Fig. 6 F). In contrast, monocyte LFA-1 and MAC-1 expressions were unaffected by CBD treatment (Fig. 6 C, D, G, and H). Taken together with the chemotaxis results, these findings suggest that THC not only inhibits monocyte chemotaxis but also modulates the conformational state of key surface integrins by reducing the expression of their active forms.

### THC Pre-Treatment Suppresses Monocyte Expression of Cytoskeletal F-actin

Actin polymerization, wherein monomeric G-actin is assembled into F-actin, is essential for spatial organization and directed chemotactic cellular movement (Hwang et al. 2024; Weiner et al. 1999). In addition, actin polymerization promotes high-affinity surface integrin conformations, in a process termed inside-out signaling (Pang et al. 2023). Inside-out integrin signaling refers to intracellular signaling events that trigger conformational changes of integrins. Furthermore, actin polymerization plays a key role in stabilizing integrins in their high-affinity state, facilitating inside-out signaling. Therefore, we additionally assessed the effects of short-term THC and CBD exposures on monocyte actin polymerization using an F-actin tracking stain (Fig. 6I and J). Short-term treatment of monocytes with THC, but not CBD, significantly reduced the expression of F-actin on a per-cell basis (Fig. 6I and J). Consistent with this finding, treatment with 10µM THC significantly decreased overall monocyte polymerized actin.

## Discussion

THC and CBD, two major cannabinoids found in *Cannabis sativa*, are well recognized for their immunosuppressive properties. Specifically, THC and CBD have been evidenced to attenuate inflammatory cytokine production from CD16^+^ monocytes, a monocyte subset implicated in crossing the BBB and contributing to neuroinflammation in HAND (Fischer-Smith et al. 2001; Rizzo et al. 2018, 2019b; Sermet et al. 2025). Furthermore, up to 50% of people with HIV still experience some level of HAND, despite effective viral suppression with cART (Cysique et al. 2004; Heaton et al. 2010). Clinical studies report that cannabis use is associated with reduced CSF levels of neuroinflammatory chemokine MCP-1, supporting the need for further investigation of cannabis and major cannabinoids as a potential therapeutic strategy to limit monocyte contributions in neuroinflammation (Watson et al. 2021). To explore the therapeutic potential of cannabinoids in managing monocyte induced inflammation, the present study examined how THC and CBD influence CD16^+^ monocyte-initiated astrocyte inflammatory responses and monocyte migration, using a primary human coculture model of CD16^+^ monocytes and astrocytes in combination with assessments of chemotaxis. We have demonstrated that both THC and CBD suppressed CD16^+^ monocyte-induced production of inflammatory mediators IL-6, IL-8, and MCP-1 from astrocytes. Additional studies established that monocytes isolated from HIV+ individuals displayed enhanced chemotaxis towards conditioned media from astrocytes treated with recombinant IL-1ß relative to their HIV- counterparts. Pre-treatment of monocytes with THC resulted in reduced chemotaxis in both donor groups, while CBD had no measurable effect. Moreover, short-term exposure to THC also decreased F-actin levels and reduced surface expression of the active conformations of LFA-1 and MAC-1. While both cannabinoids dampened CD16^+^ monocyte-initiated inflammatory cytokine production from astrocytes in coculture, only THC additionally impaired monocyte chemotactic responses.

Under normal conditions, astrocyte produced inflammatory mediators, such as IL-6, IL-8, and MCP-1, exert neuroprotective effects by maintaining CNS homeostasis (Capogna et al. 2023; Choi et al. 2014; Kim et al. 2014; Kummer et al. 2021). However, if persistently elevated, these same mediators can become neuroinflammatory and disrupt neuronal function (Conductier et al. 2010; Kummer et al. 2021; Sokolova et al. 2009). Furthermore, IL-6 functions primarily as a pro-inflammatory cytokine that directly promotes inflammatory signaling. In contrast, MCP-1 and IL-8 are chemokines, which recruit monocytes and neutrophils, respectively, to the site of infection (Bickel 1993; Deshmane et al. 2009; Mukaida et al. 1998; Tanaka et al. 2014). Notably, peripheral blood CD16^+^ monocytes have been implicated in neuroinflammatory processes across various neurodegenerative conditions, such as HAND, multiple sclerosis, and Alzheimer’s disease (Clay et al. 2007; Fischer-Smith et al. 2001; Muñoz-Castro et al. 2023; Tian et al. 2025). Therefore, elucidating how THC and CBD regulate CD16^+^ monocyte-induced astrocyte inflammatory responses can have broad implications.

Results from this current study indicated that TLR7 and TLR8 activation of cocultures significantly induced the secretion of IL-6, IL-8, and MCP-1. Because these measurements were taken from coculture supernatants, the cellular source of the mediators was initially unclear. However, the incorporation of parallel monoculture samples demonstrated that astrocytes produced substantially more IL-6, IL-8, and MCP-1 compared to CD16^+^ monocytes, suggesting that the astrocytes are the primary contributors to mediator production under coculture conditions (Figs. 2 and 4). This finding is consistent with the design of the coculture, wherein the astrocytes are seeded in a 20:1 ratio relative to the monocytes. Furthermore, both THC and CBD significantly suppressed TLR7-induced release of IL-6, IL-8, and MCP-1 from cocultures (Fig. 1). In TLR8-activated cocultures, THC and CBD also suppressed supernatant release of inflammatory mediators, although CBD did not significantly affect IL-8 secretion.

Given that previous neutralization studies have identified monocyte-derived IL-1ß as an inducing factor of astrocyte inflammatory mediators, CD16^+^ monocyte monoculture supernatant IL-1ß levels were quantified (Rizzo et al. 2019a). Consistent with previously published work from our laboratory, TLR7 and TLR8 activation significantly induced CD16^+^ monocyte IL-1ß secretion, which was markedly suppressed by THC and CBD (Sermet et al. 2025). IL-1ß levels in astrocyte monoculture supernatants fell below the detection limits, indicating that astrocytes do not produce IL-1ß in this culture (data not shown). Interestingly, the concentrations of THC and CBD that suppressed monocyte IL-1ß secretion correlated with cannabinoid concentrations that inhibited IL-6, IL-8, and MCP-1 in coculture supernatants, suggesting that cannabinoid-mediated suppression of monocyte-derived IL-1ß may have influenced astrocyte production of inflammatory factors. This is further supported by the findings from the astrocyte monoculture results wherein recombinant IL-1ß enhanced astrocyte secretion of IL-6, IL-8, and MCP-1, and was not directly suppressed by THC and CBD. These monoculture supernatant data suggest that THC and CBD reduce inflammatory cytokine production in cocultures by inhibiting monocyte IL-1ß secretion, indirectly suppressing astrocyte secretion of inflammatory factors.

Under normal conditions, the BBB restricts the entry of peripheral immune cells into the CNS. However, under neurodegenerative conditions, such as HAND, BBB integrity can be disrupted, facilitating immune cell infiltration and exacerbating neuroinflammation (Bush et al. 1999; Kim et al. 2014). Moreover, post-mortem brain samples obtained from HIV+ subjects diagnosed with HAND demonstrate peripheral CD16^+^ monocyte infiltration into the CNS (Clay et al. 2007; Fischer-Smith et al. 2001). Having demonstrated that CD16^+^ monocytes induced MCP-1 secretion from astrocytes in coculture, we next investigated whether conditioned media from IL-1ß-stimulated astrocytes would trigger monocyte chemotaxis, and whether migratory capacity would differ based on HIV status. Monocytes from both HIV- and HIV+ subjects exhibited enhanced chemotaxis towards astrocyte-conditioned media regardless of whether astrocytes were stimulated with recombinant IL-1ß, compared to the fresh media control. In contrast, recombinant MCP-1 alone was insufficient to trigger migration, which indicates that astrocyte-conditioned media likely contain a combination of soluble factors that together generate a more potent migratory signal. Interestingly, monocytes from HIV+ donors exhibited enhanced chemotaxis towards IL-1ß-treated astrocyte-conditioned media relative to their HIV- counterparts. These findings differ from earlier findings by Eugenin and colleagues, who observed robust chemotaxis toward MCP-1 in monocytes acutely infected with HIV ex vivo, which was attributed to elevated CCR2 expression (Eugenin et al. 2006). Taken together, our data instead suggest that in the context of chronic HIV infection, monocytes appear primed to respond to multifactorial signals released by activated astrocytes rather than to a single chemokine, such as MCP-1.

Given that THC and CBD have been demonstrated to inhibit the inflammatory contribution of monocytes on astrocyte responses, we considered whether they could additionally regulate monocyte migration. THC pre-treatment significantly suppressed the capacity of monocytes to migrate in response to astrocyte-conditioned media in a comparable magnitude in both HIV + and HIV- monocytes, whereas CBD had no effect. While this chemotaxis assay did not directly model BBB-mediated transmigration, it provides a controlled system to assess cannabinoid effects on chemotactic behavior. Accordingly, we examined cytoskeletal and integrin-dependent mechanisms that mediate both chemotaxis and transmigration across the BBB. Consistent with the chemotaxis assay findings, assessments of cytoskeletal polymerized actin and high-affinity conformational states of LFA-1 and MAC-1 revealed that pre-treatment with THC impaired monocyte expression of key molecular processes required for chemotaxis. The findings from this study differ, in part, from those reported by Raborn et al., who used microscopy-based approaches to demonstrate that THC inhibited Tat-induced U937 monocyte adhesion to extracellular matrix proteins by reducing ß1 integrin-expression and altering F-actin distribution (Raborn et al. 2014). Notably, in the absence of Tat, THC treatment alone increased overall ß1 integrin expression while suppressing F-actin levels in U937 monocytes (Raborn et al. 2014). An important distinction between the studies by Raborn et al. and our study lies in the integrin populations examined and the methods used to assess them. While Raborn et al. evaluated total ß1 integrin expression, the present study focused on high-affinity conformational states of LFA-1 and MAC-1, ß2 integrins that play critical roles in monocyte adhesion and migration (Li et al. 2018; Raborn et al. 2014; Sumagin et al. 2010). Given that integrin-mediated adhesion is controlled both by surface expression as well as activation state, selective assessment of high-affinity LFA-1 and MAC-1 may account for the apparent differences between our findings and those reported by Raborn et al. (Li et al. 2017; Takagi et al. 2002). Interestingly, despite differences in experimental context and integrin readouts, both studies indicate that THC alters integrin-associated adhesion mechanisms and F-actin dynamics. Based on the findings from this current study, we hypothesize that THC pre-treatment may suppress monocyte transmigration across the BBB.

Actin polymerization and integrin activation states are interconnected through a process termed “inside-out integrin signaling”, wherein intracellular signaling pathways induce conformational changes of integrins (Springer and Dustin 2012; Zhu et al. 2008). Actin polymerization is critical for stabilizing integrins in their high-affinity state and can promote inside-out integrin signaling (Zhu et al. 2008). Based on the results from this current study, it remains unclear whether THC suppresses LFA-1 and MAC-1 high-affinity states indirectly by inhibiting actin polymerization or whether it affects both processes independently. Nevertheless, these findings suggest that even a brief, 1-hour pre-treatment with THC was sufficient to diminish monocyte responsiveness to migratory cues, indicating that short-term exposure to THC can rapidly alter monocyte cytoskeletal dynamics and integrin activation. In contrast, CBD treatment did not affect monocyte chemotaxis, actin polymerization, or integrin expression. These results highlight clear immunopharmacological differences between these cannabinoids and point to a selective role for THC in suppressing monocyte chemotactic function.

THC primarily binds CBR2 to elicit its immunomodulatory effects however, can also engage with additional targets, including proliferator-activated receptor gamma (PPAR-γ), GRP55, and transient receptor potential V2 (TRPV2) (Adams et al. 1940; Devane et al. 1988; Lauckner et al. 2008; Munro et al. 1993; O’Sullivan et al. 2005; Vara et al. 2013; Zhang et al. 2022). In contrast, CBD has low binding affinity for the CBRs, and its molecular targets remain widely debated (de Almeida and Devi 2020; Kaplan et al. 2008). Nonetheless, studies have implicated fatty acid amide hydrolase, PPAR-γ, TRPV1, and adenosine A_2A_ receptors as targets by which CBD mediates its effects on the immune system (Bisogno et al. 2001; De Petrocellis et al. 2008; Mecha et al. 2013; Rodrigues et al. 2024; Watanabe et al. 1996). While THC and CBD share some overlapping targets, they may act through distinct mechanisms, which explains why their immune-modulating effects throughout this current study are similar in some respects yet divergent in others. Furthermore, previously published work from our laboratory has also identified differences by which THC and CBD suppress CD16^+^ and CD16^−^ monocyte secretion of IL-1ß, TNF-α, and IL-6 (Sermet et al. 2025). Regardless, this current study demonstrated that THC and CBD treatments may have therapeutic value in attenuating monocyte-initiated or enhanced astrocyte inflammatory responses. While both cannabinoids reduced monocyte-initiated astrocyte IL-6, IL-8, and MCP-1 secretion, only THC additionally inhibited monocyte chemotaxis, suggesting that THC may exert broader immune-regulating effects compared to CBD in the context of monocyte-mediated neuroinflammation. Additionally, through our previous findings, we identified that THC-mediated effects on monocyte inflammatory mediator secretion, including IL-1ß, are CBR2-independent (Sermet et al. 2025). In this context, we speculate the indirect effects of THC and CBD observed in the present investigation on astrocyte inflammatory cytokine secretion through inhibition of monocyte-derived IL-1ß may similarly reflect CBR2-independent mechanisms, although should be directly assessed in future investigations. It would also be valuable for future studies to assess whether any cannabinoid-mediated effects involve non-CBR mechanisms, such as PPAR-γ and TRP channels, especially given their role in regulating IL-1ß maturation and secretion (Hong et al. 2015; Scheraga et al. 2016; Wu et al. 2023; Yang et al. 2021).

Importantly, the concentrations of THC and CBD utilized throughout these studies are physiologically relevant to human exposure. A controlled human inhalation study following a strict smoking protocol and rigorous sampling revealed that after smoking, peak plasma concentrations of THC can reach an average of 152 µg/L (0.5µM), which falls within our tested concentrations (Grotenhermen 2003; Huestis et al. 1992; Perez-Reyes et al. 1982). Notably, this peak concentration of THC was observed after smoking research-grade cannabis, which is estimated to have THC content of less than 3.55% THC per dry weight. Furthermore, the THC content of research-grade cannabis is substantially lower than the average THC content found on the market today, which can reach levels as high as 30% THC, thus indicating the relevance of the higher concentrations utilized throughout this currently study. While peak plasma concentrations of CBD following cannabis use are not well studied, one study reported concentrations reaching 191 µg/L (0.6µM) after participants smoked a cannabis cigarette containing 20 mg CBD (Ohlsson et al. 1986). However, a large proportion of commercial CBD-containing products are intended for oral administration, which results in significant first-pass metabolism. Pharmacokinetic studies conducted for the FDA-approved CBD therapeutic, Epidiolex^®^ reported an average peak plasma concentration of 330 µg/L (1µM) CBD following a 20 mg/kg/day oral dosing regimen (FDA 2018). It is possible that individuals using low-dose THC- and CBD-containing products may not achieve plasma concentrations assessed within this current study. Nevertheless, due to the lipophilicity of THC and CBD, repeated or chronic exposure can result in tissue accumulation, wherein these concentrations may be reached over time.

We acknowledge that the outlined studies had several limitations. First, the coculture system utilized represents a simplified model that does not capture the full cellular complexity of the brain, which also includes microglia, pericytes, neurons, and brain microvascular endothelial cells. This specific coculture was used to examine how THC and CBD influence CD16^+^ monocyte contributions to astrocyte inflammatory responses. Future studies will need to incorporate a more complex multicellular culture that better mimics the brain environment to define CD16^+^ monocyte contributions to neuroinflammation as a whole. Additionally, the chemotaxis assay employed was not intended to model BBB transmigration. While THC clearly altered monocyte chemotactic responses, these results do not directly address its effects on monocyte recruitment to the CNS from the periphery. Additionally, we acknowledge the lack of access to donor demographic information for the HIV- control population, wherein potential contributors to observed inter-donor variability in cytokine responses, including differences in age, sex, or other biological factors could not be evaluated in the present study. We also note that HIV- and HIV+ participants in this study were recruited from different geographic locations. Finally, we recognize that the overall sample sizes within these studies were modest. However, the effects observed with cannabinoid treatments were relatively consistent across donors despite this limitation.

Overall, this study used a primary human in vitro model to expand the current body of knowledge on CD16^+^ monocyte-mediated astrocyte inflammatory responses and the potential immunomodulatory roles of THC and CBD. CD16^+^ monocytes induced astrocyte secretion of inflammatory IL-6, MCP-1, and IL-8 in TLR7- and TLR8-activated primary human cocultures. Furthermore, treatment of activated cocultures with THC and CBD inhibited the astrocyte inflammatory response indirectly by attenuating monocyte IL-1ß secretion. Additionally, we observed that monocytes from HIV+ donors exhibited enhanced chemotaxis toward astrocyte-conditioned media compared to their HIV- counterparts. Chemotaxis was diminished by short-term pre-treatment of monocytes with THC and was associated with reduced expression of F-actin and active conformations of integrins, LFA-1 and MAC-1. Taken together, this study provides evidence to support that THC, and to a lesser extent CBD, exert anti-inflammatory effects on CD16^+^ monocyte-mediated inflammatory and migratory responses that may be associated with HAND.

## Data Availability

The authors declare that all the data supporting the findings of this study are available within the paper.

## Abbreviations

CNS: Central nervous system
BBB: Blood-brain barrier
HIV: Human immunodeficiency virus
HAND: Human immunodeficiency virus-associated neurocognitive disorder
IL: Interleukin
LFA-1: Lymphocyte function-associated antigen-1
MAC-1: Macrophage-1 antigen
TLR: Toll-like receptor
TNF-α: Tumor necrosis factor-α
CSF: Cerebrospinal fluid
MCP-1: Monocyte chemoattractant protein-1
IP-10: Interferon-gamma inducible protein 10
THC: Δ^9^-tetrahydrocannabinol
CBD: Cannabidiol
CBR: Cannabinoid receptor
RPMI: Roswell Park Memorial Institute
PBMC: Peripheral blood mononuclear cells
NHA: Normal human astrocytes
PPAR: Peroxisome proliferator-activating receptor
TRPV: Transient receptor potential vanilloid
